# Induction of Proteases in Peritoneal Carcinomatosis, the Role of ICAM-1/CD43 Interaction

**Published:** 2007-10-08

**Authors:** Nawar A. Alkhamesi, Gretta Roberts, Paul Ziprin, David H. Peck

**Affiliations:** Professor Sir Ara W. Darzi KBE, MD, FRCS, FRCSI, FACS, FRCPSG, FMedSci, Department of Biosurgery and Surgical Technology, Imperial College London, U.K

**Keywords:** Colorectal cancer, CD43, Heparin, ICAM-1, MMP-2 and MMP-9, Mesothelial cells

## Abstract

**Introduction:**

The development of peritoneal metastases is a significant clinical issue in the treatment of abdominal cancers and is associated with poor prognosis. We have previously shown that ICAM-1-CD43 interaction plays a significant role in tumor adhesion. However, an invasive phenotype is critical to establish tumor progression via cell associated and secreted proteases including matrix metalloproteinases. High metalloproteinases level significantly enhanced metastasis phenotype on tumors, a detrimental effect on surgical outcome. We investigated the role of direct and indirect signaling between the mesothelium and the tumor cells in enhancing tumor invasion and possible therapeutic intervention.

**Methods:**

Mesothelial cells were enzymatically derived from human omental tissue and implanted in 24 wells plates. Colorectal cancer cells were then introduced and allowed a direct and an indirect contact with the mesothelial layer. Anti-ICAM antibodies, anti-CD43 antibodies, and heparin were used to block MMP production. Gelatin zymography was performed on the supernatant to detect MMPs activity.

**Results:**

MMP production was observed in mesothelial and tumor cells. Direct contact between cell types enhanced MMP9 and 2 (p < 0.05). Indirect contact also stimulate MMPs but at a lower degree. ICAM-1 blocking antibodies attenuated MMP production in direct contact to that observed in the indirect. Heparin introduction achieved a similar outcome.

**Conclusions:**

ICAM-1-CD43 interaction plays a vital role in tumor cells-peritoneum adhesion and invasion, which is manifested by the increased production of MMPs leading to tumor invasion and peritoneal loco-regional. Blocking this interaction with heparin can provide a new therapeutic option.

## Introduction

Colorectal cancer is the most common gastrointestinal cancer in the U.K. and U.S.A. It is estimated that 30,000 new cases will be diagnosed in the U.K. and up to 150,000 in the U.S.A. each year (Cancer Research U.K., 2003; Jemal et al. 2003). Early detection and surgery remain the only curative treatment. However, even with surgery and adjuvant therapy, the five year survival is around 60% (O’connell et al. 2004).

Nearly one third of patients with colorectal malignancies suffer from metastases at the time of diagnosis and about 50% who have early stage cancer may develop metastases (Midgley and Kerr, 1999; Coutinho and Rocha Lima, 2003). In general, patient with peritoneal metastases have a poor prognosis (Sugerbaker et al. 1996) and out of those who undergo curative operations, around 25% could develop peritoneal recurrence (Brodsky and Cohen, 1991; Gunderson et al. 1985; Pestieau and Sugarbaker, 2000; Piso et al. 2007; Sugarbaker, 2007).

In order for peritoneal growth to happen, the tumor cells have to adhere to the mesothelium and/or the extracellular matrix, proliferate and then invade the submesothelial space to gain access to the circulation in order to achieve distance metastases (Schlaeppi et al. 1997). A number of cell adhesion molecules have been implicated to play a vital role in the adhesion process such as CD44, β1 integrin, ICAM-1 and CD43 (Alkhamesi et al. 2005b; Schlaeppi et al. 1997; Ziprin et al. 2004). However, induction of invasion is not clearly defined. The invasive process requires local proteolysis of the extracellular matrix, pseudopodial extension, and cell migration (Woodhouse et al. 1997). Both normal and malignant cells are capable of invading tissue because they secret matrix metalloproteinases (MMPs) that degrade the extracellular matrix basement membrane (Aoudjit et al. 1998).

Matrix metalloproteinases are a family of functionally related zinc-dependent proteinases that are capable of degrading all the components of the extracellular matrix. This group includes gelatinases, collagenases and stromelysins (Li et al. 2002; Pross et al. 2002). The expression of MMPs is closely regulated by activating agents, including cytokines (IL-2, IL-4) and cell adhesion molecules of the integrin family (VCAM-1, ICAM-1) (Aoudjit et al. 1998). Moreover, their expression has been correlated with the invasive phenotype in a variety of human malignancies, including breast, colorectal, hepatocellular, lung and prostate cancers (botos et al. 1996). Blocking MMPs activity specially MMP-2 and MMP-9 has been implicate to inhibit tumor invasion and angiogenesis (Albini et al. 1991; Schnaper et al. 1993; Brown, 1997).

In this study, we investigate the effect of CD43-ICAM-1 interaction on MMP-2 and MMP-9 activities and the possibility of a therapeutic intervention.

## Methods

### Cell lines

Human primary mesothelial cells were obtained from omental samples taken from patients undergoing elective abdominal operations, a procedure approved by St. Mary’s Hospital NHS Trust ethical committee. The cells were isolated as described by Stylianou et al. (Stylianou et al. 1990). Briefly, the omental samples were digested in 0.25% trypsin (Life Technologies, Paisley, U.K.) for 20 min, on a tube rotator, at room temperature and then centrifuged at 800 g for six minutes. The pellet was then suspended in Media 199 with Earle’s balanced salts and L-glutamine containing 10% fetal calf serum (FCS) Life Technologies, Paisley, U.K.) supplemented with penicillin/streptomycin 100 μgml^−1^, insulin 5 μgml^−1^, apo-transferrin 5 μgml^−1^, hydrocortisone 0.4 μgml^−1^ (All Sigma Chemical Co., U.K.) and grown to sub-confluence in 25 cm^2^ culture flasks at 37 °C in air with 5% CO_2_ and a complete changeofmediaevery2–3days. Immunocytochemical and morphological analysis of the mesothelial cells confirmed purity of cell cultures. Positive staining for cytokeratin 18 and vimentin (both Chemicon, U.K.) together, with absence of von Willebrand Factor (CN Biosciences, U.K.), differentiated these cells from fibroblasts and endothelial cells, respectively. The mesothelial cells used for each experiment were at passage two.

The colorectal cell line (SW1222) used were obtained from the European Collection of Cell Cultures (ECACC, CAMR, Wiltshire, U.K.) and grown to sub-confluence in 75 cm^2^ culture flasks using DMEM with glutamax (Life Technologies, Paisley, U.K.) supplemented with 10% fetal calf serum (Life Technologies, Paisley, U.K.) at 37 °C in air with 5% CO_2_ and complete change of media every 2–3 days.

### Cell viability and proliferation

Cell viability and proliferation was assessed in all cell lines after subjection to the various models. This was accomplished using a non-radioactive cell proliferation assay (Cell Titer 96 Aqueous Non-Radioactive Cell Proliferation Assay; Promega, U.K.). Absorbance of this product was measured by a photospectometer (Titertek Multiskan MKII, Titertek, U.K.) at 490 nm.

### Co-culture assay

Mesothelial cells were seeded at a density of 1.5 × 10^6^ cells per well of a flat-bottomed 24-well plates (Helena Bioscience, Sunderland, U.K.) in supplemented Media 199 with Earl’s salt and cultured to sub-confluence. The media was then removed and the cells were washed twice with serum free media for 30 mins. Tumor cells were detached with non-enzymatic cell dissociation solution (Sigma Chemical Co., U.K.), and washed twice at 37 °C in serum free media. Tumor cells (1.5 × 10^4^ cells in 0.5 ml of serum free Media 199) were added to wells containing mesothelial cells, either in direct contact or separated by a 3.0 μm pore size filter (Corning Costar, U.S.A.) and incubated at 37 °C for 24 hrs.

For inhibition studies, anti-ICAM and anti-CD43 antibodies where included in the appropriate wells at a previously determined concentration of 20 μgml^−1^ simultaneously with the tumor cells (Ziprin et al. 2004). In order to determine the effect of heparin on MMPs expression in the co-culture system, the assays were repeated with mesothelial cells which had been pre-treated with 100 iu/ml heparin (Alkhamesi et al. 2005b) and the tumor cells re-suspended in the same heparin containing medium. After 24 hrs, the supernatants were collected and levels of MMP-2 and MMP-9 activity determined using gelatin zymography.

### Gelatin zymography

MMP-2 and MMP-9 activity of cell culture supernatants was determined as described by Aoudjit et al. (Aoudjit et al. 1997). Briefly, supernatant were centrifuged at 1200 rpm for 10 min to remove contaminating cells and debris, and the supernatants subjected to electrophoresis on a 12% SDS-PAGE containing 1 mgml^−1^ gelatin. After electrophoresis, the gel was washed in 2.5% Triton X-100 to remove the SDS and incubated in developing buffer (50 mM Tris, 5 mM CaCl_2_, 0.02% NaN_3_, 1% Trion X-100) for 18 hrs at 37 °C. Lyric bands were visualized by staining with Coomassie brilliant blue and de-stained in 30% ( v/v) methanol and 10% ( v/v) acetic acid. Quantitative analysis of activity was counted using computerized densitometry imager (Nonlinear Dynamic, Nonlinear U.S.A. Inc., U.S.A.).

## Results

### Mesothelial cells and tumor cells express MMP-2 and MMP-9

To establish a base line of MMP production in mesothelial and tumor cells, both groups were cultured separately to sub-confluence in a suitable media as described in material and methods. The media was then changed into serum free media and the cells were grown for further 24 hrs. The supernatants were collected and tested for MMP-2 and MMP-9.

**Figure 1:**
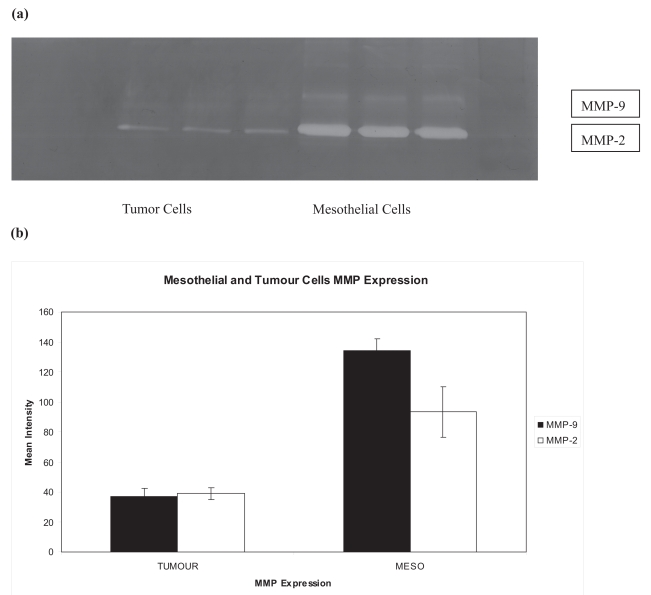
Mesothelial and Tumor cells MMP expression. **(a)** Characteristic gelatin zymography indicating both mesothelial and colorectal tumor cells expression MMP2 and MMP9. Proteases activity is higher in the mesothelial cells. Each line indicates one of triplicate taken from either SW1222 or primary mesothelial cells. **(b)** Graph shows the results of minimum three experiments.

MMP-2 expression was stronger in the z cell line in comparison to SW1222, P = 0.05. MMP-9 activity was also higher in mesothelial cell and almost negligible in SW1222 cell line, P = 0.05 (Fig. 1).

### Tumor-mesothelial cells interaction up-regulate MMPs expression

To study the effect of direct and indirect contact between mesothelial cells and colorectal tumor cells, both cell lines were co-cultured together for 24 hrs either with direct contact or separated by a 3.0 μm pore size filter. The supernatants were collected and studied for MMP-2 and MMP-9 productions.

**Figure 2:**
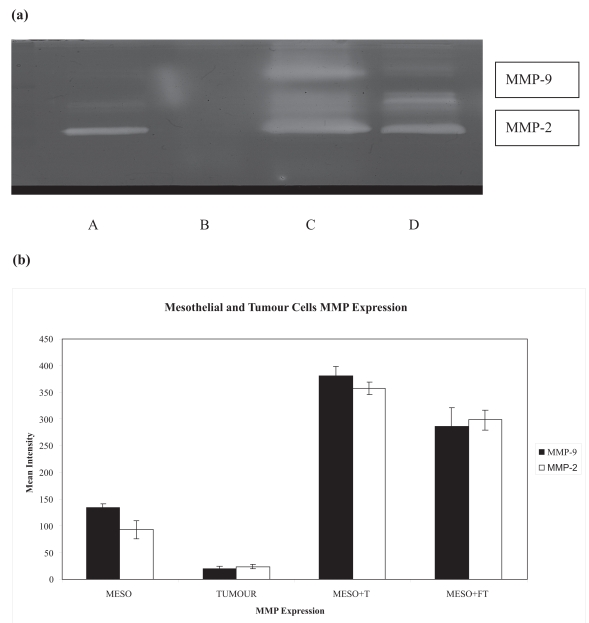
Mesothelial-Tumor cells interactions up-regulate MMP expression. **(a)** Representative gelatin zymography shows total MMP-2 and 9 productions by mesothelial cells (A) and tumor Cells (B). Mesothelial cells in direct contact with tumor cells (C) and in. indirect contact with tumor cells (D). There is clear up-regulation of MMPs expression when the mesothelial cells were co-cultured with tumor cells. MMPs expression by tumor cells alone in negligible. **(b)** Graph indicating the results of minimum three experiment (Meso + FT = Mesothelial cell + Filter + Tumor cells. Meso + T = Mesothelial cells + Tumor cells).

Mesothelial cells showed constitutive expression of MMP-2, no significant up-regulation was observed when they were allowed indirect contact with tumor cells, however, noticeable increase in MMP-2 activity occurred when direct contact was initiated, P = 0.05. This was accompanied by the induction of MMP-9 activity P = 0.05 (Fig. 2). These data imply the role of direct contact in the initiation of protease production and therefore the possible involvement of cell adhesion molecules.

### ICAM-1 and CD43 blockage attenuate tumor-mesothelial MMPs expression

**Figure 3:**
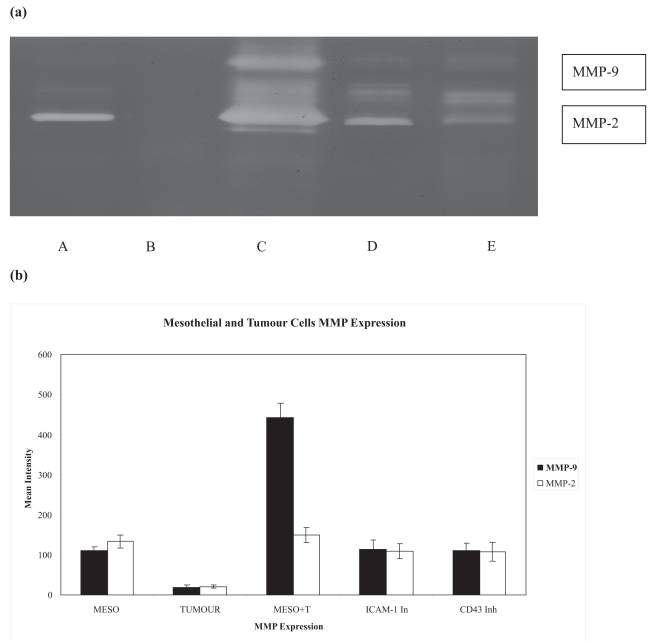
Blocking cell adhesion molecules decreases proteases expression. **(a)** Representative gelatin zymography shows MMP-2 and 9 expressions by mesothelial cells (A) and tumor cells (B). This expression was up-regulated when both mesothelial and tumor cells co-cultured together (C). However, the introduction of anti-ICAM (D) and anti-CD43 (E) antibodies blocked this interaction. **(b)** Graph shows the results of minimum three experiments.

We can see clearly from the previous results that tumor-mesothelial cells adhesion up-regulate MMP-2 and MMP-9 expression. We have also shown previously that this adhesion is ICAM-1 dependent (Alkhamesi et al. 2005b). To investigate whether ICAM-1 and/or its ligand CD43 play a significant role in MMPs production following the mesothelial-tumor cells co-culture, ICAM-1 antibodies or CD43 antibodies were introduced to the co-culture and incubated for 24 hrs. The introduction of both sets of antibodies reduced MMP-2 and MMP-9 production in comparison to co-cultured cell lines without antibodies, P = 0.03 and 0.02 respectively (Fig. 3).

### Heparin down-regulate MMPs production following mesothelial-tumor cells interaction

The introduction of heparin to mesothelial-Tumor cells interaction has successfully blocked adhesion by down-regulating ICAM-1 expression (Alkhamesi et al. 2005b). We examined the possibility of introducing heparin to the tumor-mesothelial co-culture to attenuate MMPs production.

Heparin was added to the co-culture and the cell lines were incubated for further 24 hrs. The supernatants were collected and a gelatin zymography was performed to measure MMP-2 and MMP-9 production.

**Figure 4:**
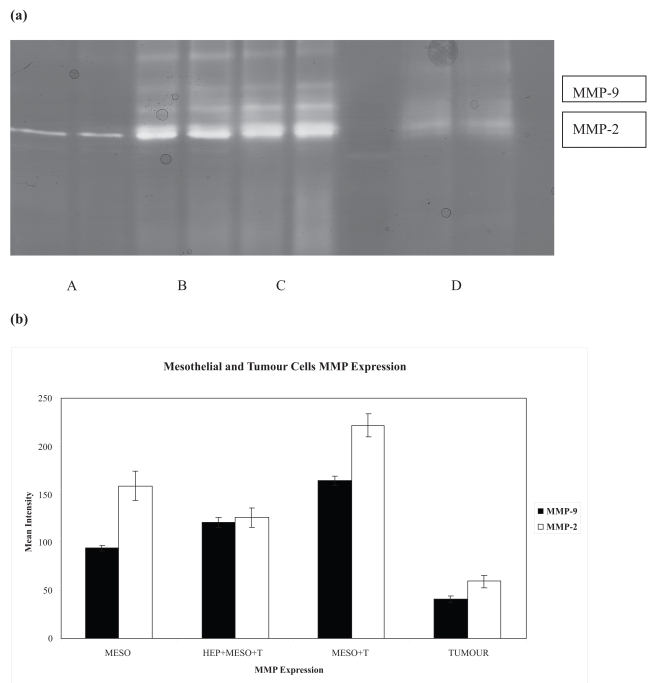
Heparin reduces proteases production. **(a)** Representative gelatin zymography shows MMP-2 and 9 expressions by mesothelial cells (A) and tumor cells (D). Heparin down-regulate MMP-2 and 9 activities when it was introduced (B) in comparison to the standard co-culture (C). **(b)** Graph indicating the results of minimum three experiments.

It is clear from Figure 4 that heparin introduction had decreased MMP2 and MMP-9 production in comparison to control co-culture, P = 0.04 and 0.03 respectively.

## Discussion

The productions of MMPs have been proposed as a mechanism by which tumor cells can penetrate and invade target tissue. They are up-regulated in almost every human cancer and usually associated with poor prognosis (Baker et al. 2006). MMPs function by cleaving a diverse group of substrates that include in addition to the structural components of the extra-cellular matrix, growth-factor-binding proteins, growth-factor precursors, receptor tyrosine kinases, and cell adhesion molecules (Egeblad and Werb, 2002).

In previous studies, we showed that heparin could successfully down-regulate ICAM-1 production *in vitro* resulting in preventing tumor adhesion. Subsequent tumor growth and metastases were significantly reduced in *in vivo* animal models (Alkhamesi et al. 2005b; Alkhamesi et al. 2005a). These *in vivo* data implied more than a simple onestep block of adhesive function and suggested a possible role in the reduction of invasion capacity.

ICAM-1 has been shown to initiate malignant invasion in a number of malignancies via its interaction with apical MUC-1 on circulating tumor cells and this interaction can be enhanced by tumor cytokine activities (Roland et al. 2007). It also documented that inhibition of ICAM-1 production by host and/or tumor cells can decrease MMP activity resulting in reduction of adhesion and subsequent invasion (Hyoudou et al. 2007)

In this study, we demonstrated that MMP-2 and MMP-9 are produced predominantly by mesothelial cells and their expression is up-regulated once there is a contact between the mesothelial cells and the tumor cells. This up-regulation is more prominent with direct contact indicating the important role of cell adhesion molecules and subsequent signaling. These data also show the vital role that ICAM-1 plays not only in initiating tumor-mesothelium adhesion, but also in preparing the right environment for subsequent invasion despite some published data suggesting that the up-regulation of ICAM-1 inhibit tumor progression and indicate a better prognosis (Maeda et al. 2002; Tachimori et al. 2005)

Previously published data have shown bi-directional signaling during contact between tumor cells and target tissue, in particular lymphoma cells and the endothelial cells can result in high expression of MMP-2 and MMP-9 in both cell types (Aoudjit et al. 1997). These results are similar to what we have demonstrated in the current study in which the introduction of tumor cells into the co-culture triggered a higher expression of MMP-2 and MMP-9 via a bi-directional signaling.

Blocking this vital cell adhesion molecule (ICAM-1), by using inhibiting antibodies clearly reduced MMP-2 and MMP-9 production and offers possible new generation of therapeutics that can target both adhesion and invasion.

By applying heparin to this experimental design, we were able to achieve similar results to blocking ICAM-1 and CD43. These finding support our previous results and enforce the important role that heparin can play in the surgical management of intra-abdominal cancers to prevent loco-regional peritoneal metastases.

## Conclusions

We have shown that bi-directional signaling between mesothelial and tumor cells is an important factor in generating cancer invasion and that ICAM-1/CD43 interaction is vital to this phenomenon. We also confirmed the role of heparin as a potential successful therapeutic in preventing postoperative peritoneal loco-regional metastases.
